# Enhancing radiation tolerance by controlling defect mobility and migration pathways in multicomponent single-phase alloys

**DOI:** 10.1038/ncomms13564

**Published:** 2016-12-15

**Authors:** Chenyang Lu, Liangliang Niu, Nanjun Chen, Ke Jin, Taini Yang, Pengyuan Xiu, Yanwen Zhang, Fei Gao, Hongbin Bei, Shi Shi, Mo-Rigen He, Ian M. Robertson, William J. Weber, Lumin Wang

**Affiliations:** 1 Department of Nuclear Engineering and Radiological Sciences, University of Michigan, Ann Arbor, Michigan 48109, USA; 2 Materials Science and Technology Division, Oak Ridge National Laboratory, Oak Ridge, Tennessee 37831, USA; 3 Department of Materials Science and Engineering, University of Tennessee, Knoxville, Tennessee 37996, USA; 4 Department of Engineering Physics, University of Wisconsin-Madison, Madison, Wisconsin 53706, USA; 5 Department of Materials Science and Engineering, University of Michigan, Ann Arbor, Michigan 48109, USA

## Abstract

A grand challenge in material science is to understand the correlation between intrinsic properties and defect dynamics. Radiation tolerant materials are in great demand for safe operation and advancement of nuclear and aerospace systems. Unlike traditional approaches that rely on microstructural and nanoscale features to mitigate radiation damage, this study demonstrates enhancement of radiation tolerance with the suppression of void formation by two orders magnitude at elevated temperatures in equiatomic single-phase concentrated solid solution alloys, and more importantly, reveals its controlling mechanism through a detailed analysis of the depth distribution of defect clusters and an atomistic computer simulation. The enhanced swelling resistance is attributed to the tailored interstitial defect cluster motion in the alloys from a long-range one-dimensional mode to a short-range three-dimensional mode, which leads to enhanced point defect recombination. The results suggest design criteria for next generation radiation tolerant structural alloys.

## Abstract (web summary)

Radiation tolerance is a property determined both by materials structure and defect dynamics. Here authors demonstrate enhancement of radiation tolerance at elevated temperatures in equiatomic single-phase concentrated solid solution alloys and propose an underlying mechanism.

The safety and advancement of nuclear and aerospace technologies rely on catapulting beyond current knowledge and incremental property improvements in radiation tolerant material^1^,^2^,^3^,^4^. Introducing high-densities of defect sinks, such as secondary phase or grain boundaries, is a popular way for reducing residual defects in irradiated materials. The success of this approach has been demonstrated in oxide-dispersion-strengthened steels^5^,^6^,^7^, nano-layered^8^ and nano-grained polycrystalline alloys^9^. However, the nanostructures in the above materials are often unstable at elevated temperatures and in extreme radiation environments^10^,^11^. On the other hand, some previous studies have suggested that tuning the composition of a single-phase binary alloy may also enhance the radiation tolerance of the material significantly. For example, Wang *et al*.^12^ found that increasing the Cu content (10–50 at.%) in Ni–Cu alloys may effectively suppress irradiation-induced void swelling at the peak swelling temperature^12^. Despite these reports, debates on the long-term effectiveness of mechanisms to control or enhance radiation tolerance of single-phase alloys continue.

A class of materials called single-phase concentrated solid-solution alloys (SP-CSAs), including high-entropy alloys (HEAs), have received much attention recently due to their unique structures and excellent properties. In contrast to conventional alloys, SP-CSAs are composed of two to five-principal elements in equal or near-equal molar ratios that form random solid solutions in either a simple face-centred cubic (f.c.c.) or simple body-centred cubic (b.c.c.) crystal lattice structure. The complicated random arrangement of alloying elements and local chemical environment at atomic level lead SP-CSAs to exhibit extraordinary properties compared with traditional alloys, such as high thermal stability and hardness, high strength-to-weight ratio, high-temperature strength, great wear and fatigue resistance, and also excellent corrosion resistance^13^,^14^,^15^,^16^. In addition, the high-level site-to-site lattice distortions and compositional complexities in SP-CSAs can effectively reduce the mean free path of electrons, phonons and magnons; these distortions and complexities can also be used to modify formation energies, migration barriers and diffusion pathways of irradiation-induced defects, thereby modifying defect generation, interaction, interstitial–vacancy recombination in the early stages of irradiation^17^,^18^,^19^,^20^. A recently published paper indicates that equiatomic alloys may be more resistant to radiation damage than the corresponding pure elements^21^. To obtain a full picture of the fundamental controlling mechanism on enhanced radiation tolerance, partly due to the development of defect clusters at elevated temperature and high irradiation doses, a set of SP-CSAs with different alloying elements needs to be investigated. Understanding the performance of SP-CSAs under high-dose irradiation at elevated temperatures provides a scientific foundation for designing radiation tolerant materials.

In this study, nickel and five Ni-containing equiatomic SP-CSAs, NiCo, NiFe, NiCoFe, NiCoFeCr and NiCoFeCrMn, were irradiated with 1.5 and/or 3 MeV Ni^+^ ions to two different ion fluences at 773 K. Nickel and NiFe were irradiated with ions of both energies. The irradiated microstructures were all characterized by cross-sectional transmission electron microscopy (TEM). The resulting microstructure can be qualitatively differentiated into two distinct groups based on the characteristics of the surviving defect clusters and their depth distribution. One group (nickel and NiCo) shows large voids (vacancy clusters) in the irradiated region that are distributed closer to the sample surface, but in front of the interstitial-type dislocation structures. The other group (NiFe, NiCoFe, NiCoFeCr and NiCoFeCrMn) contains much smaller voids distributed at the very end of the ion range, deeper in depth than the interstitial-type dislocation loops that appear in the irradiated region. Increasing the ion energy and fluence moved the defect clusters deeper and enlarged the cluster size but did not change the fundamental characteristics of the defect distribution. The unique depth distribution of vacancy and interstitial clusters in equiatomic SP-CSAs irradiated at the elevated temperature is associated with a much more significant difference between radiation tolerance among the alloys, that is, a two orders of magnitude enhancement at 773 K versus a factor of 2–4 enhancement at the room temperature^17^.

## Results

### Enhanced void swelling resistance in SP-CSAs

**Figure 1:**
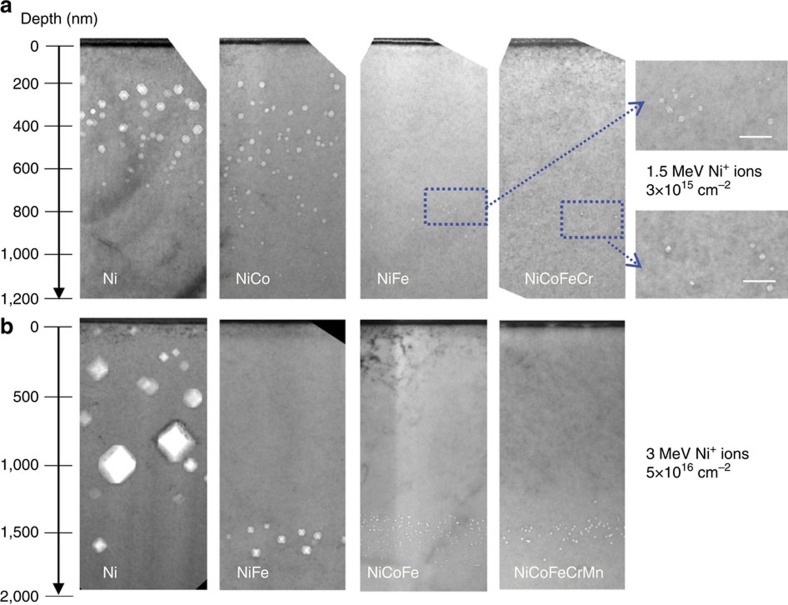
Irradiation-induced void distribution. (**a**) Cross-sectional TEM images of nickel, NiCo, NiFe and NiCoFeCr irradiated with 1.5 MeV Ni^+^ ions to 3 × 10^15^ cm^−2^ at 773 K, scale bars in the zoomed images are 50 nm. (**b**) Cross-sectional TEM images of nickel, NiFe, NiCoFe and NiCoFeCrMn irradiated with 3 MeV Ni^+^ ions to 5 × 10^16^ cm^−2^ at 773 K. The ions enter the specimen from the top of the images.

For the 1.5 MeV Ni-irradiated samples, the predicted damage range is ∼800 nm and the peak damage level is ∼4 dpa (displacement per atom) with an ion fluence of 3 × 10^15^ cm^−2^, as calculated using the Stopping and Range of Ions in Matter 08 code (SRIM 08) in Kinchin–Pease mode with a displacement threshold energy of 40 eV (ref. 22). The predicted profiles are shown in Supplementary Fig. 1a. Although all four materials (pure nickel, NiCo, NiFe and NiCoFeCr) irradiated at 773 K have non-negligible void distributions in or out of the predicted damage range, as shown in the bright-field cross-sectional TEM images in Fig. 1a, the much smaller voids in NiFe and NiCoFeCr represent much higher swelling resistance than in nickel and NiCo. The swelling values represent the local volume changes due to the void formation, which is calculated by a common equation^23^. While nickel shows a significant overall swelling of ∼1.8% and NiCo has a lower void swelling of about 0.42%, NiFe and NiCoFeCr exhibit substantially lower void swelling with similar values of ∼0.02%, more than two orders of magnitude lower than in nickel.

Figure 1b shows the cross-sectional TEM images of four samples (pure nickel, binary alloy NiFe, ternary alloy NiCoFe and HEA NiCoFeCrMn) irradiated with 3 MeV Ni^+^ ions at 773 K to an ion fluence of 5 × 10^16^ cm^−2^ corresponding to a peak damage dose of 60 dpa Again, nickel shows the highest swelling with an overall swelling of 9.4%. NiFe shows a significantly improved swelling resistance, with a swelling value of 0.45%. NiCoFe and NiCoFeCrMn show even smaller swelling, ∼0.15% and 0.1%, respectively. It is noted that similar discrepancies of the void depth distribution in nickel and NiFe are observed in both cases. The void distributions in NiCoFe and NiCoFeCrMn are similar to NiFe, except the voids are even smaller. Summarizing the results from the two ion irradiation experiments, the relative swelling resistance of these six materials can be ranked in the order from the worst to the best as nickel<NiCo<NiFe≤NiCoFeCr<NiCoFe≤NiCoFeCrMn.

### Void-interstitial loop distribution

**Figure 2:**
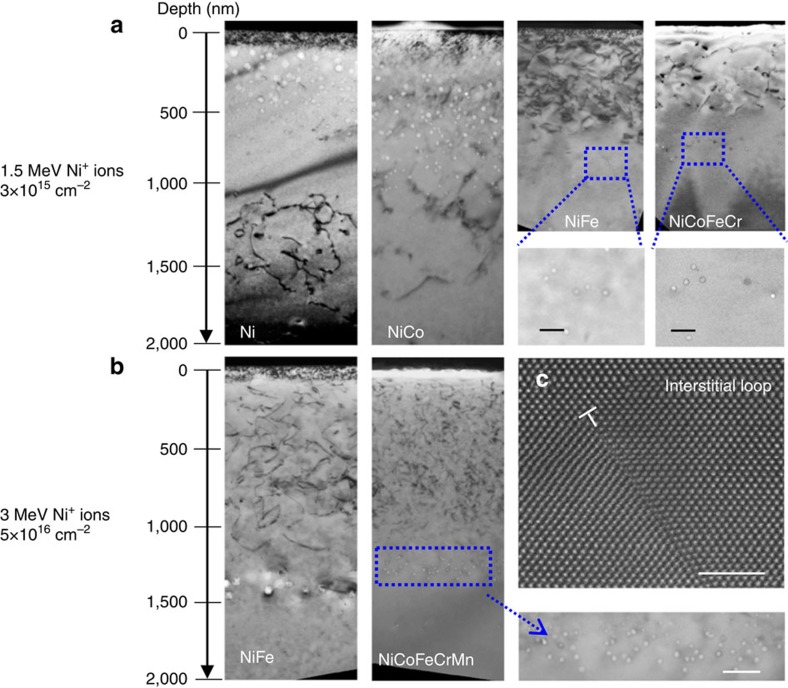
Distribution of dislocations and dislocation loops in nickel and Ni-containing SP-CSAs. (**a**) Cross-sectional TEM images of nickel, NiCo, NiFe and NiCoFeCr irradiated by 1.5 MeV Ni^+^ to 3 × 10^15^ cm^−2^ at 773 K, scale bars in the zoomed images are 50 nm. (**b**) Cross-sectional TEM images of NiFe and NiCoFeCrMn irradiated by 3 MeV Ni^+^ ions to 5 × 10^16^ cm^−2^ at 773 K, scale bar in the zoomed image is 100 nm. **a**,**b** were taken under the two-beam condition with *g*=200. (**c**) High-resolution high-angle annular dark field STEM image showing a part of an interstitial loop in NiCoFeCr; scale bar, 2 nm.

While voids are the result of three-dimensional (3D) agglomeration of irradiation-induced vacancies, dislocation loops and network dislocations are often the most typical defects formed in irradiated materials due to the agglomeration of interstitials^24^. Figure 2a shows bright-field cross-sectional TEM images from nickel, NiCo, NiFe and NiCoFeCr irradiated with 1.5 MeV Ni^+^ to 3 × 10^15^ cm^−2^ at 773 K. The images were all taken under the same two-beam condition, with *g*=[200], for imaging the dislocation structures. Obviously, voids and dislocations distributed separately along the depth in the four materials, and this separation presents two scenarios.

First, as shown in Fig. 2a, network dislocations, including long dislocation lines and larger dislocation loops are found at a deeper depth than the voids in nickel and NiCo. Second, NiFe and NiCoFeCr show the opposite behaviour in defect cluster distribution, as revealed in Fig. 2a. Large dislocation loops are found near the sample surface, extending from the surface to a depth of 600 nm followed by a low density of small voids. Higher-magnification bright-field TEM micrographs of dislocation structures of four materials are shown in Supplementary Fig. 2. A higher density of smaller voids was found to exist further from the surface than the dislocation loops in NiFe and NiCoFeCr. In addition, similar dislocations and void distributions were observed in NiFe and NiCoFeCrMn irradiated with 3 MeV Ni^+^ ions to 5 × 10^16^ cm^−2^, as shown in Fig. 2b.

The most interesting observation of this study is that the void and dislocation structures were heterogeneously distributed in two unique ways across the depth range among the six f.c.c. alloys. During high-temperature irradiation at 773 K (∼0.45 of the melting temperature), irradiation-induced vacancies are mobile^24^. The supersaturated concentration of vacancies can agglomerate together to form voids or vacancy type of dislocation loops, while interstitial defects can agglomerate into dislocation loops that grow into network dislocation lines. Careful characterization of the dislocation loops using the inside–outside contrast method^25^ (Supplementary Fig. 3), as well as high-resolution high-angle annular dark field scanning TEM (STEM; Fig. 2c), confirmed the interstitial nature of the dislocation loops observed. Apparently, the resistance to void swelling of the SP-CSAs is related to the segregation between vacancy and interstitial types of defect clusters that is essentially the result of self-organization of irradiation-induced vacancies and interstitials.

### Theoretical defect cluster migration behaviour

One-dimensional (1D) motion of small interstitial clusters has been proposed and observed in a molecular dynamics (MD) study of copper^26^,^27^. It has been demonstrated that small clusters of self-interstitial atoms can migrate one-dimensionally along the close-packed row of atoms in the lattice. In simple metals, the migration barrier of the small glissile interstitial clusters is very low for 1D motion. Thus, they can migrate extremely fast along the direction of their Burgers vector. The 1D motion of small defect clusters has also been observed directly by *in situ* TEM during irradiation^28^,^29^. Long-distance 1D migration of interstitial clusters will eliminate interstitials for recombination in the local area due to a variety of mechanisms by allowing the interstitials to reach distant regions or sinks, leaving a high-vacancy supersaturation behind and, thus, leading to significant void swelling. On the basis of the TEM results of this study, we propose that the 1D motion of interstitial defects dominates in nickel and NiCo, but has apparently been suppressed in NiFe, NiCoFe, NiCoFeCr and NiCoFeCrMn by the existence of complexity alloying effects on defocusing 1D motion.

**Figure 3:**
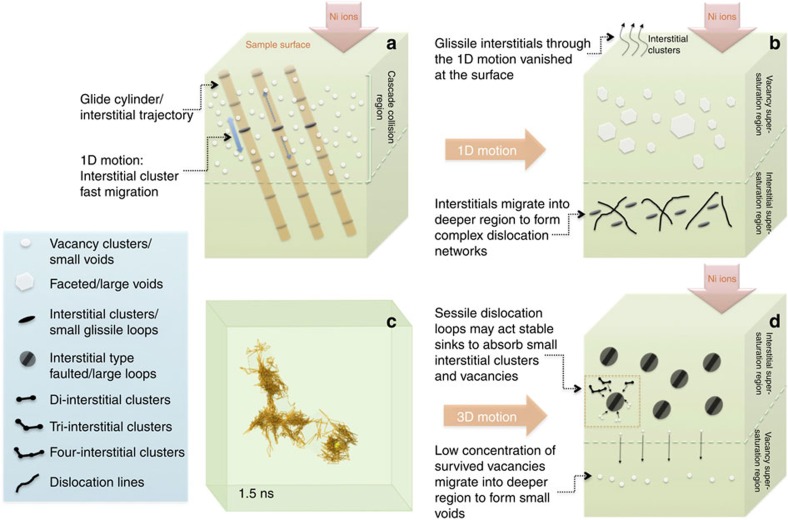
1D and 3D motions of interstitial clusters under ion irradiation. (**a**) Schematic illustration of 1D motion in nickel and NiCo. Interstitial clusters migrated fast along the glide cylinder. (**b**) Schematic sketch of defect evolution and distribution in nickel and NiCo as a result of **a**. (**c**) MD simulation result of the trajectory of the centre of a four-interstitial cluster in NiFe showing a 3D migration mode. (**d**) Schematic sketch of defect evolution and distribution in NiFe, NiCoFe, NiCoFeCr and NiCoFeCrMn as a result of **c**.

MD simulations have been carried out to interpret the experimental observations of this study. The calculated diffusion coefficients of single interstitial and small interstitial clusters (I_1_–I_4_) for NiFe are shown in Supplementary Fig. 4a. The defect migration energy barriers (*E*_m_) and exponential pre-factors (*D*_0_) in NiFe are shown in Table 1, along with those in pure nickel for comparison. The *E*_m_ for a single interstitial in NiFe is higher than that in pure nickel^30^,^31^, which may be attributed to the difference in atomic size between nickel and iron, as well as the structural distortions in the random solid solution alloy. It is of interest to note that the *E*_m_ of interstitial clusters in NiFe increases with increasing cluster size, in contrast to that in nickel, where the *E*_m_ of interstitial clusters is almost independent of the cluster size. The key difference between NiFe and nickel is that the small interstitial clusters migrate randomly in 3D in NiFe, but exhibit 1D migration in nickel. As schematically illustrated in Fig. 3a, if an interstitial cluster migrates along the glide cylinder with 1D motion in pure nickel, while the vacancy concentration in the cylinder is equal to that in the environment, the interstitial cluster can migrate a long distance without encountering and sweeping up a significant number of vacancies. Small interstitial clusters in nickel moved quickly from the damage production region to the surface and to deeper regions, leaving a high-vacancy supersaturation behind to be susceptible to detrimental void formation. A fraction of the glissile interstitial clusters migrated to the deeper region and eventually grew into network dislocations as shown in Fig. 3b.

**Figure 4:**
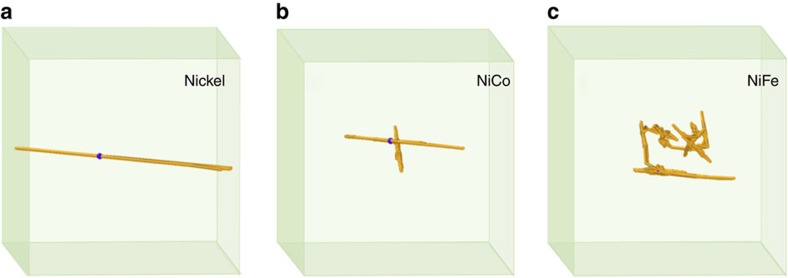
Trajectories of the centre of a nine-interstitial cluster. (**a**) In nickel at 800 K, (**b**) in NiCo at 1,200 K and (**c**) in NiFe at 1,200 K. The trajectories clearly show the different migration behaviours in these alloys.

However, as shown in the MD simulation results of Fig. 3c, the trajectory of the mass centre for a four-interstitial cluster in NiFe clearly migrates in a 3D mode at 1,100 K. Although the cluster can occasionally move along one direction, the diffusion segment is too short to be characterized as 1D migration. Also, the migration behaviours of nine-interstitial clusters are simulated for 20 ns at 1,200 K for NiFe and NiCo, but at 800 K for nickel because the interstitial clusters in NiFe and NiCo have much higher migration energy barriers due to the existence of different species, thus moving much slower than in nickel at the same temperature. The migration trajectories of the cluster centres are shown in Fig. 4. It can be clearly seen that the interstitial cluster in nickel migrates only one-dimensionally along the 〈110〉 direction. The interstitial clusters in NiFe and NiCo also migrate mainly along the 〈110〉 direction, they randomly change directions, thus forming a number of 1D 〈110〉 segments (Supplementary Movies 1–3). However, the 〈110〉 migration segments in NiCo are, on average, much longer than that in NiFe. Six changes in direction appear within 20 ns in NiFe, but only one in NiCo. The average distance of the 〈110〉 segments over 20 ns is about 1 nm for NiFe, but 3 nm for NiCo at 1,200 K. The shorter 1D migration segment and more frequent direction changes of interstitial clusters in NiFe will eventually lead to a dominant 3D migration behaviour, as shown in Fig. 3c. The different behaviours of interstitial clusters between NiFe and NiCo may account for the different void distribution and the swelling resistance in these alloys.

### *In situ* observation of defect cluster migration

**Figure 5:**
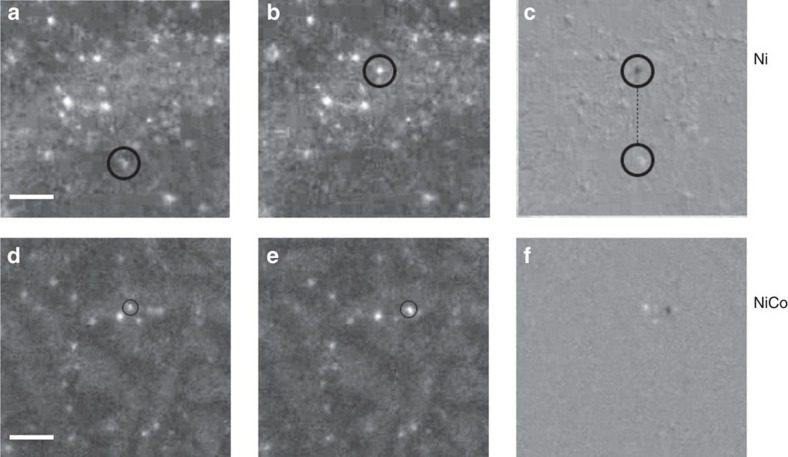
1D glide of interstitial clusters in nickel and NiCo. Observations were conducted by *in situ* TEM. (**a**) The initial position of the dislocation loop in nickel, (**b**) the final position of the loop in nickel, (**c**) superimposing image of the initial and final position of loop in nickel, (**d**) the initial position of the loop in NiCo, (**e**) the final position of the loop in NiCo and (**f**) superimposing image of the initial and final position of loop in NiCo; scale bar, 20 nm.

On the basis of TEM results shown in Fig. 1, it is reasonable to believe that the 1D migration of interstitial clusters dominates in nickel and NiCo, but 3D motion of interstitial clusters dominates in NiFe and other SP-CSAs. This assumption has not only been verified by the MD simulation mentioned above but has also been demonstrated by our *in situ* TEM observation of dislocation loop migration under 1 MeV Kr^+^ ion irradiation at 773 K with the IVEM-Tandem Facility at Argonne National Laboratory. Small loops, presumed to be interstitial in character, glided further than large interstitial loops. The frequency of occurrence of 1D glide, as well as the glide distance both decreased with increasing alloy complexity. In comparing the two binary alloys, the frequency of 1D glide was higher and occurred over longer distances in the NiCo than in NiFe. An example of 1D glide in nickel and NiCo is shown in the weak-beam dark-field TEM micrographs presented in Fig. 5, consistent with the result of the MD simulations. The 1D migration of interstitial clusters has not been observed in more complex HEAs.

## Discussion

At first glance, it seems that the atomic size difference of the component elements of the alloys may be a simple criterion. The different atomic sizes of elements in these SP-CSAs contribute to enhanced atomic scattering and, thus, lead to a degradation of focused motion of interstitials along the close packed direction and a suppression of 1D mode, which prevents the interstitials from migrating rapidly out of the vacancy concentrated region in NiFe, NiCoFe, NiCoFeCr and NiCoFeCrMn. Therefore, defect recombination in these alloys is promoted. The atomic volume size factors of specific alloying elements deviating from the atomic volume size of nickel in the solid solutions are shown below. Much larger volume deviations from Ni are found in Cr (+10.34%), Fe (+10.57%) and Mn (+23.2%) than in Co (+1.76%)^32^. However, more careful exploration reveals that the insight behind the superficial size effect can be directly related to a more complex effect correlated to the substantial reduction of electron, phonon and magnon mean free paths, which control the complex defect formation energy and migration barrier landscapes^17^. Furthermore, high-level lattice distortion can not only reduce the defect mobility but also provide effective trapping for the annihilation of freely migrating defects^33^,^34^,^35^. As shown in the schematic image of Fig. 3d, sessile interstitial loops can absorb vacancies, therefore decreasing the vacancy concentration in the matrix and effectively suppressing void formation and swelling in the alloys. The 3D migration of interstitial clusters against 1D migration may provide one of the mechanisms accounting for the enhanced radiation resistance of NiFe, NiCoFe, NiCoFeCr and NiCoFeCrMn.

On the other hand, vacancies could also migrate by thermal activation and this did indeed occur in all materials^29^,^31^. Some vacancies migrate to the sample surface where they are annihilated, and others migrate into deeper regions of the material. Owing to the increased interstitial concentration thus higher probability for recombination in the deeper region of nickel and NiCo, vacancies could not aggregate to form voids there. However, in NiFe, NiCoFe, NiCoFeCr and NiCoFeCrMn, most of the interstitial clusters were immobile and remained in the defect production region, leading to a higher defect recombination rate. As shown in Fig. 3d, only the vacancies that escaped from the cascade formation range have the opportunity to agglomerate into voids in the deeper regions, while most vacancies are likely annihilated by interstitials in the shallower region. Thus, compared with the voids found in nickel and NiCo, only smaller voids are observed in the deeper regions of NiFe, NiCoFe, NiCoFeCr and NiCoFeCrMn. Among these four materials, the HEA NiCoFeCrMn clearly shows the best swelling resistance probably due to the most lattice distortion caused by the atomic volume size deviation. These results demonstrate that tuning compositional complexity in these concentrated single-phase alloys can significantly improve the irradiation performance by modified defect cluster motion mode.

The diffusion coefficients of single vacancies and vacancy clusters as a function of reciprocal temperature are plotted in Supplementary Fig. 4b, and their corresponding migration energy barriers and exponential pre-factors are displayed in Table 1. The migration energy barrier of a single vacancy (V_1_) in NiFe is about 0.76 eV, slightly lower than that in nickel, which may again be associated with the size difference of the atoms in the alloy. A striking feature in the results of the present MD simulations is that the migration energy barriers of vacancy clusters are smaller than that of a single vacancy, particularly for a di-vacancy with migration energy of 0.515 eV. Di- and tri-vacancy clusters have migration energy barriers similar to that for a single interstitial, resulting in interstitials and vacancies migrating simultaneously, without a temperature window to control the migration of different defects. On the basis of the fundamental theory of defect evolution (Supplementary Note 1), the similar mobility of interstitials and vacancies in most equiatomic SP-CSAs may lead to a significantly higher recombination rate, resulting in much lower concentration of surviving defects in the matrix. This feature is significantly different from that in pure nickel, where interstitials migrate much faster than vacancies^31^, and the majority of vacancies remain in the crystal matrix, leading to significant void formation at elevated temperature.

The integrated experimental and modelling work shows that reduced defect mobility and altered migration paths on a modified energy landscape, resulting from alloy complexity, can modify defect dynamics to promote annihilation of radiation damage that ultimately enhance radiation tolerance. Irradiation-induced void formation in nickel and Ni-containing equiatomic SP-CSAs at elevated temperature is controlled by the migration behaviours of interstitial clusters. Short-range 3D motion of interstitial clusters that significantly increases the vacancy–interstitial recombination rate plays a key role in preventing significant void swelling in NiFe, NiCoFe, NiCoFeCr and NiCoFeCrMn. The mechanism has been revealed by TEM observation and confirmed by MD simulation. Composition complexity and lattice distortion due to the alloy components effectively modify the defect migration behaviour in the equiatomic SP-CSAs. More localized migration of interstitial clusters enhances recombination of point defects in the cascade region where the defects are created. Tuning the compositional complexity of concentrated solid-solution alloys has been shown to be an effective approach for developing a new generation of radiation-tolerant materials.

## Methods

### Ni ion irradiation

Mechanically polished nickel, NiCo, NiFe and NiCoFeCr were irradiated with 1.5 MeV Ni^+^ ions to fluences of up to 4 × 10^14^ and 3 × 10^15^ cm^−2^ at 773 K in the Ion Beam Materials Laboratory at University of Tennessee. Another batch of samples, including nickel, NiFe, NiCoFe and NiCoFeCrMn were irradiated with 3 MeV Ni^+^ ions to fluence of 5 × 10^16^ cm^−2^ at 773 K. Raster beam was conducted to obtain a homogeneous irradiation. Predicted local dose and implanted Ni ion concentration in all of the samples were calculated by the SRIM 2013 code under option of quick mode with a displacement threshold energy of 40 eV, as shown in Supplementary Fig. 1a,b.

### Defect characterization

FIB lift-out techniques were used for TEM samples preparation by using an FEI Helios Nanolab workstation. Under-focused bright-field images were performed for characterizing void distributions. TEM thin foils were tilted away from the zone axis for emphasizing the void contrast. A flash polishing was conducted to remove FIB-induced damage that might be confused with the damage from the primary ion irradiation. A double Cs-corrected S/TEM JEOL 3100R05 was used for both TEM and STEM imaging under two modes. Bright-field TEM images were taken at an exposure time of 2 s by a high-speed charge-coupled device camera with 2,048 × 2,048 pixels. STEM images were taken with an inner angle of 59 mrad and camera length of 15 cm. The image pixels and exposure time/pixel for imaging in STEM were 2,048 × 2,048 and 20 μs, respectively.

### Molecular dynamics

To understand the experimental observations, we have also carried out MD computer simulations for the migration of defects and defect clusters in NiFe and NiCo (both vacancies and interstitials) with defect cluster size up to 9. At a given temperature, the trajectories of single interstitial and interstitial clusters can be tracked during MD simulations. Given enough simulation time, the mean square displacement (MSD) analysis calculated with ‘trajectory time decomposition' technique can be used to extract reliable values of the diffusion coefficient, *D*, and then the migration energy can be obtained by the Arrhenius equation^36^. The interatomic interactions of Fe–Fe, Ni–Ni and Fe–Ni are described using the embedded atom method potential developed by the Bonny *et al*.^37^, while Co–Co, Ni–Ni and Co–Ni are described by an modified embedded atom method potential developed by Kim *et al*.^38^. The potentials used present f.c.c. phase as the most stable phase in both NiFe and NiCo. For diffusion of defects and small defect clusters, an MD box of 10*a*_0_ × 10*a*_0_ × 10*a*_0_ with 2,000 Ni and 2,000 Fe/Co is used, where *a*_0_ is the lattice constant of a perfect f.c.c. structure (3.5339 Å for NiFe and 3.60 Å for NiCo). However, a larger MD box containing 32,000 atoms is used to simulate the migration of a nine-interstitial cluster. The time step is chosen to be 1 fs with the NVT ensemble (number of atoms, volume and temperature remain constants), with simulation time up to ∼20 ns, and temperature varies from 800 to 1,200 K. The defect properties in NiFe and NiCo are sampled in 10 different random structures. Within 20 ns simulation, defect clusters can migrate several hundred nanometres, which should sample many different chemical environments. However, it should be noted that the average binding energy per vacancy to a vacancy cluster is about 0.4 eV per atom, which suggests that the vacancy clusters will dissociate at high temperatures. For example, a di-vacancy cluster dissociates into two single vacancies at about 1.3 ns at 1,100 K, but it is observed that the di-vacancy makes significant jumps before dissociation. Only MSDs before dissociation are used, but we have carried out at least 10 simulations for each temperature and each vacancy cluster, starting from different equilibrium configurations. Then, the MSD averaged over all the simulations is used to determine the diffusion coefficients of the vacancy clusters.

### Data availability

The authors declare that all data supporting the findings of this study are available from the corresponding author on request.

## Additional information

**How to cite this article:** Lu, C. *et al*. Enhancing radiation tolerance by controlling defect mobility and migration pathways in multicomponent single-phase alloys. *Nat. Commun.* **7,** 13564 doi: 10.1038/ncomms13564 (2016).

**Publisher's note:** Springer Nature remains neutral with regard to jurisdictional claims in published maps and institutional affiliations.

## Supplementary Material

Supplementary Information Supplementary Figures 1 - 4, Supplementary Note 1 and Supplementary References

Supplementary Movie 1 Showing 1-D migration of a nine-interstitial cluster at 800 K in nickel. The long-range motion of the interstitial cluster are simulated with the boundary conditions in a MD box, and thus, the cluster can disappear through one face of the MD box, and reenter into the simulation box through another face.

Supplementary Movie 2 Showing 1-D migration segments along the <110> direction of a nine-interstitial cluster at 1200 K in NiCo, where the total simulation time is 20 ns.

Supplementary Movie 3 Showing 1-D migration segments along the <110> direction of a nine-interstitial cluster at 1200 K in NiFe, where the total simulation time is 20 ns.

## Figures and Tables

**Table 1:** Migration energy barriers *E*_m_ and exponential pre-factor *D*_0_ of point defects (vacancy and interstitial) and their clusters in NiFe as compared with that in pure nickel^30^,^31^,^39^.

Cluster	*E*_m_ (eV)		*D*_0_ (cm^2^ s^−1^)	
	NiFe	Nickel	NiFe	Nickel
I_1_	0.516	0.16, 0.1	0.0046	3.1 × 10^−4^
I_2_	0.617	0.12	0.0152	—
I_3_	0.728	0.04	0.0475	—
I_4_	0.974	0.002	0.3482	—
V_1_	0.756	1.32	0.0024	0.005
V_2_	0.515	0.9	0.0016	2.3 × 10^−4^
V_3_	0.624	—	0.0179	—
V_4_	0.747	—	0.0035	—
