# Supplementary material for: Enhancing radiation tolerance by controlling defect mobility and migration pathways in multicomponent single-phase alloys
Source: Nat Commun. 2016 Dec 15;7:13564. doi: 10.1038/ncomms13564 (PMC5171798; doi:10.1038/ncomms13564)
Supplement: Supplementary Information — Supplementary Figures 1 - 4, Supplementary Note 1 and Supplementary References [file ncomms13564-s1.pdf]

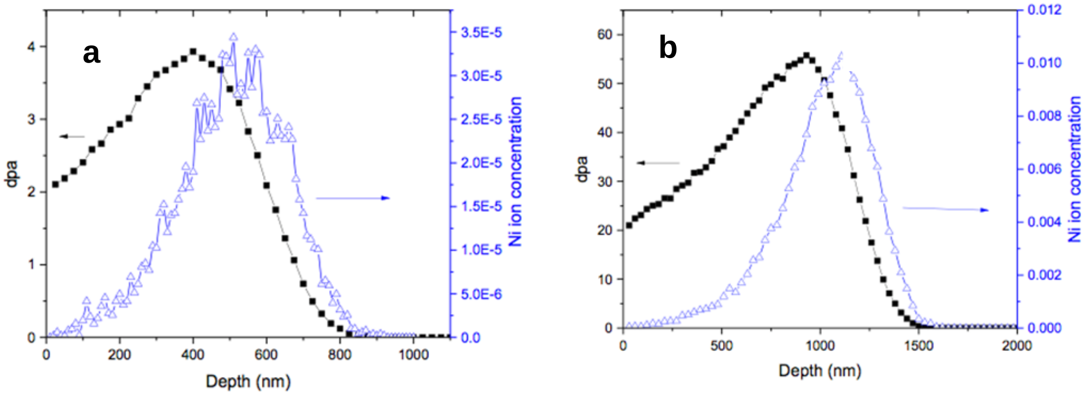


**Supplementary Figure 1 |** SRIM prediction profile of depth distribution of displacement damage and implanted ions (**a**) 1.5 MeV $Ni^+$ ions to $3\times10^{15}$ $cm^{-2}$, (**b**) 3 MeV $Ni^+$ ions to $5\times10^{16}$ $cm^{-2}$.

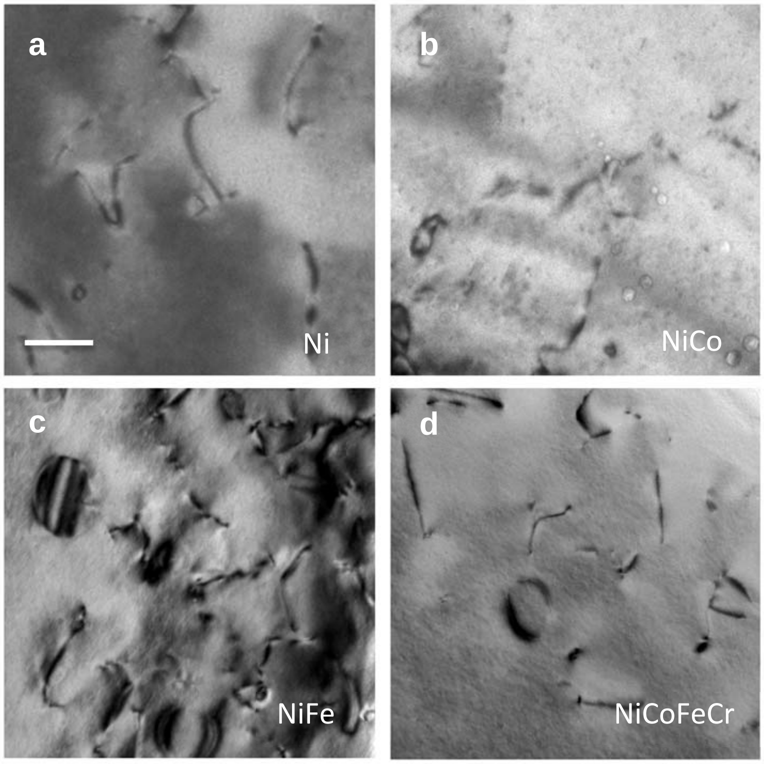


**Supplementary Figure 2 |** Dislocation structures in nickel, NiCo, NiFe and NiCoFeCr irradiated with 1.5 MeV $Ni^+$ ions to $3\times10^{15}$ $cm^{-2}$ at 773 K. (**a**) nickel, (**b**) NiCo, (**c**) NiFe, (**d**) NiCoFeCr. The scale bar is 100 nm.

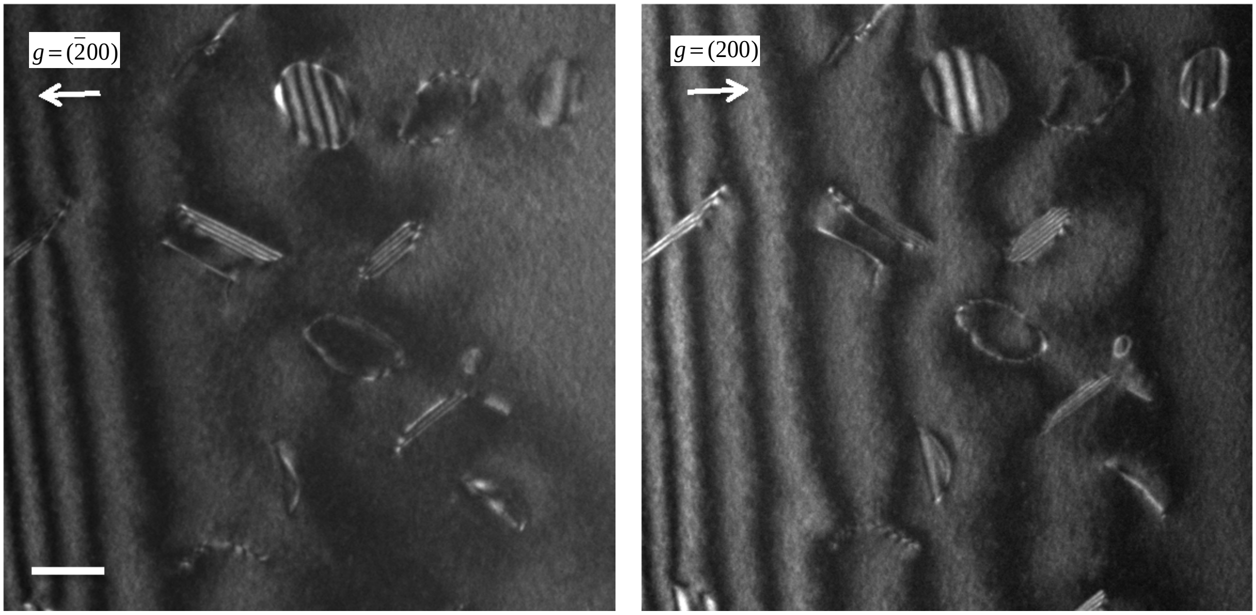


**Supplementary Figure 3 |** Characterization of the nature of dislocation loops by inside-outside method in NiCoFeCr irradiated with 1.5 $MeV^{+}$ ions to $3\times10^{15}$ $cm^{-2}$ at 773 K. Weak-beam dark field images are recorded using different **g** vectors at (**g**, 4**g**), with specimen oriented close to [011] zone axis. The dislocations are all identified as interstitial type. The scale bar is 50 nm.

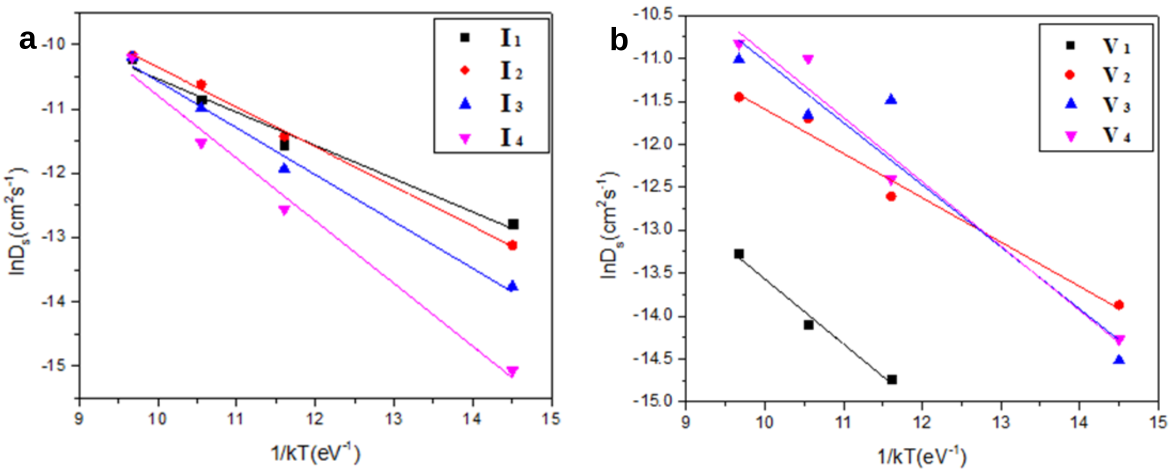


**Supplementary Figure 4 |** Self-diffusion coefficients of (**a**) interstitials and (**b**) vacancies in NiFe as a function of reciprocal temperature. The lines are drawn as a guide for the eyes. The values for the effective migration barriers and the pre-exponential factors are given in Table 1.

**Note 1: Fundamental theory of defect evolution**

In the fundamental level, displacement cascades produce interstitials and vacancies, and the concentrations of these defects under irradiation can be simply described by[1]

$$\frac{\partial C_v}{\partial t} = K_0 - K_{iv}C_iC_v - \sum K_{vs}C_vC_s$$

$$\frac{\partial C_i}{\partial t} = K_0 - K_{iv}C_iC_v - \sum K_{is}C_iC_s$$

where $C_v$ is vacancy concentration, $C_i$ interstitial concentration, $K_0$ defect production rate, $K_{iv}$ vacancy-interstitial recombination rate constant, $K_{vs}$ vacancy sink strength and $K_{is}$ interstitial sink strength. Here, we consider only single phase crystal without sinks (such as GBs, dislocations and precipitates), and thus, $C_s = 0$. $K_{iv}$ is proportional to the interstitial and vacancy diffusion coefficients, $D_i$ and $D_v$. In metals, $D_i$ is much larger than $D_v$ and thus, interstitial migration controls the microstructural evolution under irradiation. For example, the $D_i$ is $7.3\times10^{-5}$ $cm^2s^{-1}$ at 800 K in nickel, which is four orders magnitude larger than the $D_v$ ($2.2\times10^{-9}$ $cm^2s^{-1}$) [2] and the interstitials migrate very fast either to free surface or forming interstitial type dislocation loops.

**Supplementary References**


1. Was, G. S. *Fundamentals of radiation materials science: metals and alloys* (Springer, New York, 2007).
2. Zhao, L., Najafabadi, R. & Srolovitz, D. J. Determination of vacancy and atomic diffusivities in solid solution alloys. *Acta Mater.* **44,** 2737–2749 (1996).
